# Effectiveness and Efficiency of Improving HIV Service Provision for Key Populations in Nicaragua

**DOI:** 10.3389/fpubh.2016.00249

**Published:** 2016-11-16

**Authors:** Edward Ivor Broughton, Oscar Nunez, Rafael Arana, Alexey Oviedo

**Affiliations:** ^1^Research and Evaluation, USAID ASSIST Project, University Research Co., Bethesda, MD, USA; ^2^International Health Associate, Johns Hopkins Bloomberg School of Public Health, Baltimore, MD, USA; ^3^PrevenSida Project, USAID, University Research Co., Managua, Nicaragua

**Keywords:** vulnerable populations, HIV infections, Nicaragua, cost efficiency analysis

## Abstract

**Objective:**

HIV in Nicaragua is concentrated among key populations (KPs) – men who have sex with men, female sex workers, and female transgender – in whom prevalence is 600–4,000 times higher than the general population. The United States Agency for International Development PrevenSida project is aimed at increasing healthy behavior among KPs and people with HIV and improving testing, counseling, and continuity of prevention and treatment by building capacity and improving performance of non-governmental organizations (NGOs) providing services to KPs. We evaluated the cost-effectiveness of PrevenSida’s activities.

**Methods:**

This retrospective observational evaluation used individuals in KPs covered by NGOs receiving assistance from PrevenSida from 2012 to 2014. Cost-effectiveness analysis compared PrevenSida’s intervention with business-as-usual. Model inputs were generated from epidemiological modeling and PrevenSida’s records.

**Results:**

By 2014, 24 NGOs received grants and technical assistance from PrevenSida with 72,955 people in KPs served at $11.32/person ($9.39–$16.55/person, depending on region). The estimated incremental cost-effectiveness ratio was $50,700/HIV case averted or $2,600/Disability-adjusted Life Year (DALY) averted (95% CI: $1,000–$99,000 and $50–$5,100, respectively).

**Conclusion:**

PrevenSida distributed about $600,000 in grants and used $230,000 to support 24 NGOs in 2014. Cost-effectiveness from the program perspective compared to no program was slightly over half of GDP per capita per DALY averted, considered highly cost-effective by WHO criteria. Cost and efficiency varied by region, reflecting the number of people in KPs receiving services. Cost-sharing by NGOs improved cost-effectiveness from the program perspective and likely promotes sustainability. Focused interventions for KP service provision organizations can be acceptably efficient in this setting.

## Introduction

Cases of HIV in Nicaragua are concentrated among groups of individuals referred to as key populations (KPs), such as men who have sex with men (MSM), female sex workers (FSW), and female transgender people (FT). In 2013, HIV prevalence among MSM was 7.5%, among FSW was 1.9%, and among FT was 13.8% (1), whereas in the general population it was 0.003% (2).

To control the country’s epidemic, the United States Agency for International Development (USAID) Nicaragua has funded the PrevenSida Project to reach KPs through building capacity and improving performance of Nicaraguan non-governmental organizations (NGOs) that provide services to KPs. PrevenSida is a 6-year project aimed at increasing healthy behavior among those most at risk of HIV/AIDS transmission. Its goals are to strengthen institutional capacities of NGOs working with KPs, improve access to and quality of HIV/AIDS preventive services, reduce stigma and discrimination among KPs, and improve coverage of KPs by NGOs. The project grants funds to KP NGOs and works to improve data quality and continuity of care for those with HIV. To encourage sustainability, PrevenSida requires participating NGOs to use material support from sources outside the project, including in-kind donations of HIV test kits, condoms and lubricants, administrative resources use such as buildings and capital equipment, and in-kind donations of labor.

It is important for the Nicaraguan Ministry of Health, USAID, and the United States President’s Emergency Plan for AIDS Relief (PEPFAR) to know the efficiency and effectiveness of the activities implemented by PrevenSida, especially as PEPFAR pivots toward service providers in geographic areas with high burden and focuses on transparency, accountability for impact, and accelerating core interventions for epidemic control (3).

An external evaluation of bilateral USAID programing, which included assessment of the PrevenSida Project, was commissioned by USAID and conducted in 2014 (1). It showed success in capacity-building for key organizations involved with the response to HIV and good communication and coordination between them. However, there was no examination of the cost of the combination prevention model for KPs and no evaluation of efficiency of capacity development for NGOs. The current study evaluates the cost-effectiveness of the PrevenSida activities in terms of expenditure per additional KP individual receiving services from a supported NGO, per case of HIV averted, and per disability-adjusted Life Year (DALY) averted. It estimates the cost and efficiency of nationwide expansion and consolidation of this prevention approach.

### Research Questions

The questions for this evaluation are:

1. What is the cost and efficiency of the prevention program implemented by PrevenSida in terms of the projected proportion of HIV infections averted?
2. What is the cost-effectiveness, in terms of DALYs averted, of the PrevenSida intervention?

### Intervention

PrevenSida provided technical assistance using principles of quality improvement to develop human resources competencies in preventive service provision, community outreach, and general management. It worked to address accessibility gaps in KPs and developed a combined prevention model based on working with the civil society organization networks in their own social spaces and complementing public services (4).

Following project start-up in 2011, the intervention was fully underway by 2012 when 12 NGOs were receiving technical support. Capacity development for NGO management included organizing boards of directors, defining overall strategic and annual plans, developing internal accountability and budget formulation and management, and formulating overall monitoring systems. For service provision activities, a computer-based monitoring and epidemiological system (Unique Recording System) was instituted across all participating NGOs to facilitate referral of KPs to specific target services, to maximize access and coverage, and to track performance in terms of risk behavior changes (condom and lubricant use, HIV/STI testing, counseling and referral) among the targeted KPs. In 2013, the number of NGOs participating in the grants program increased to 17, and in 2014, the number increased to 24. Technical and administrative experts based in Managua traveled to all participating NGOs to provide one-on-one training, coaching, and mentorship on three to five occasions through the interventiion period. The NGOs were required to report data through the Unique Recording System, comply with administrative reporting, and remain in frequent communication throughout their involvement.

## Materials and Methods

### Study Design

This retrospective observational evaluation considered KPs covered by participating NGOs who received technical assistance from PrevenSida between 2012 and 2014. Variables of interest include the grants to each NGO for institutional strengthening and prevention activities between 2012 and 2014, the population reached with prevention services by the NGOs, the proportion of KPs reported to have changed their risk behavior, and estimated incidence of HIV in the population of interest from 2010 to 2014.

### Sampling

The study population is all of the KPs receiving services from participating NGOs in each fiscal year (FY). NGOs were included if they received grants from PEPFAR and the Key Population Challenge Fund (a financing mechanism established to expand coverage of preventive services to hidden or hard-to-reach KPs) specifically for HIV prevention activities for KPs. Data on 100% of the universe is available from those NGOs: 12 NGOs in FY 2012, 17 in FY2013, and 24 in FY2014.

### Data Collection

PrevenSida has an extensive database recording the preventive services delivered by NGOs that uses an anonymous and unique code for each service recipient to protect privacy. No additional information was required for this study – it was done entirely with the routine data collected through the Unique Recording System, including information by age, gender, population type, service received, number of contacts, geographical site where the service was delivered, and HIV test results. Other sources of information were PrevenSida’s financial records, which tracked grant payments to NGOs and staff costs for activities directly related to providing support to the NGOs. We used the project funder’s perspective for the analysis and therefore did not include the cost-sharing that was mandated for the NGOs.

Because of the anonymous nature of the data recording system and because no additional primary data were collected from clients or heath-care providers, the evaluation presented no risks to participants. The study was approved by the Centro de Investigaciones y Estudios de la Salud (CIES) of the Universidad Nacional Autónoma de Nicaragua (UNAN Managua).

### Analysis

**Figure 1:**
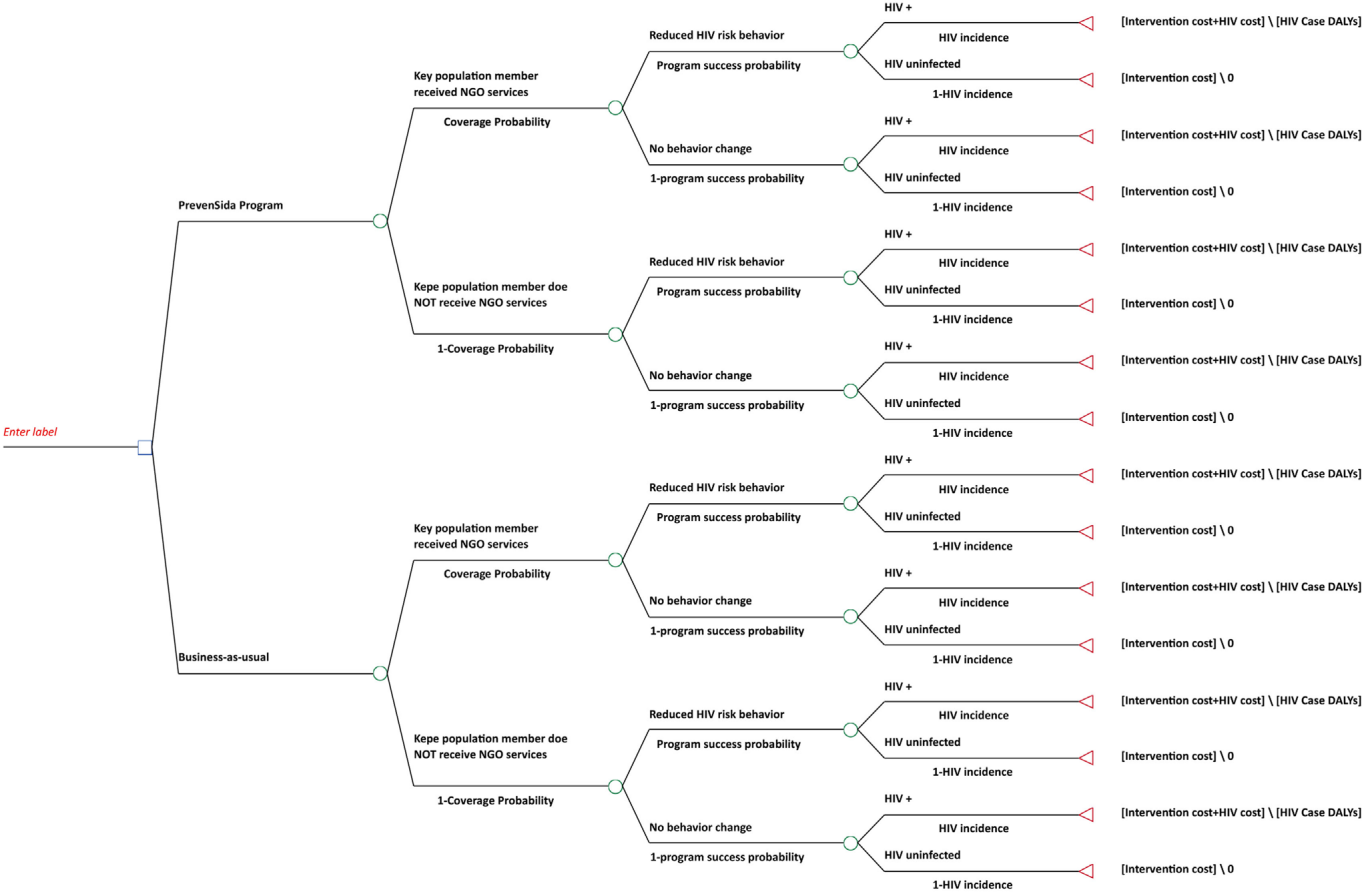
**Decision tree for the PrevenSida cost-effectiveness evaluation**.

To determine the efficiency of coverage of KPs, cost-effectiveness evaluation was conducted using decision-tree analysis comparing the PrevenSida intervention with business-as-usual (Figure 1). Inputs into the model in terms of the change in the risk of HIV were generated from the “Transmission Model” from UNAIDS (5), which used data from the PrevenSida records as inputs to estimate the number of individuals expected to develop HIV infection. Results were expressed in cost per additional person tested for HIV, cost per case of HIV infection averted, and cost per KP receiving services.

## Results

By 2014, 24 NGOs were receiving grants and technical assistance as part of their involvement in PrevenSida with a total of 74,080 people receiving their services (72,955 KPs plus 1,125 confirmed HIV cases served by them). The total cost per person in the key population reached was $11.32 with a range of $9.39–$16.55 per person depending on the region in which the NGO operated (Table 1).

**Table 1:** **Costs and coverage of NGOs by PrevenSida by region, 2014**.

Region	NGOs	PrevenSida costs	Cost per NGO ($)	Grant total ($)	Number reached		Cost per person reached
					KP (at risk)	PHIV	
Caribbean	5	70,363	14,073	133,673	12,280	49	16.55
Pacific	14	121,962	8,712	349,205	44,049	1,076	10.44
Central	4	23,454	5,864	93,979	12,510	–	9.39
RSJ	1	18,763	18,763	26,918	4,116	–	11.10

Total	24	234,542	9,773	603,775	72,955	1,125	11.32

We compared the cost of grants and administrative costs for technical assistance provided by PrevenSida between NGOs that had been working with the project for 3 years to those working for only 1 year. For comparability, they were chosen from the same regions. There was a difference in the number of people the NGOs were providing services to in the two categories, with the more experienced NGOs serving four or more times as many people in KPs. Therefore, the costs per capita for inexperienced sites were several times higher, both for the grants and for the PrevenSida administrative costs, even though the absolute costs were about half those of the experienced sites (Table 2). All costs were considered from the perspective of the funder of the PrevenSida Project.

**Table 2:** **Grants and administrative costs for selected NGOs by region**.

NGO	Region	Grants	Admin Costs	People reached	Admin cost per capita	Per capita total cost
**Experienced NGOs**						
A	Central	33,902	5,864	6,766	0.87	5.88
B	Pacific	33,502	8,712	4,695	1.86	8.99
C	Pacific	33,098	8,712	4,076	2.14	10.26
**Inexperienced NGOs**						
F	Central	16,634	5,864	1,318	4.45	17.07
D	Pacific	15,452	8,712	2,001	4.35	12.08
E	Pacific	11,074	8,712	784	11.11	25.24

The costs reported here do not include those related to the cost-sharing requirement of the participating NGOs. These amounted to $700,000 between 2012 and 2014. Approximately 40% was as in-kind donations, 28% was for administrative and capital costs, such as rental of buildings and depreciation of vehicles, and the remaining 32% was for in-kind labor from volunteer staff. Sources for cost-share resources include the Global Fund for AIDS, TB and Malaria, UNAIDS, and other multilateral donors.

The inputs for the decision-tree model used to estimate cost-effectiveness were obtained from the PrevenSida database directly or from the data used in the UNAIDS transmission model to estimate the number of new cases occurring before and after the PrevenSida intervention was operational (Table 3). These were entered into the model with binomial distributions corresponding to the degree of uncertainty.

**Table 3:** **Key epidemiological inputs for cost-effectiveness model**.

Variable descriptions		Value	Source
Probability of becoming HIV+ if risk behavior reduced, 2014	N	2,765	Comision Nicaraguense del SIDA (6)
	D	3,265,000	
Probability that KP gets NGO services, 2014	N	42,271	Comision Nicaraguense del SIDA (6)
	D	80,280	
Probability of risk behavior reduction when exposed to NGO, 2014		57%	Instituto Nacional de informacion de desarrollo (7)

Probability of HIV infection if no change in risk behavior in KP	N	3,387	Comision Nicaraguense del SIDA (6) and PrevenSIDA (8)
	D	3,265,000	
Probability of risk behavior reduction if KP not exposed to NGO, 2014		38%	Comision Nicaraguense del SIDA (6); PrevenSIDA (8), and UNAIDS (5)
			Comision Nicaraguense del SIDA (6)
Probability that KP gets NGO services, 2010	N	3,065	Comision Nicaraguense del SIDA (6) and UNAIDS (5)
	D	74,280	Comision Nicaraguense del SIDA (6)
Probability of reduced risk behavior with NGO, 2010		38%	UNAIDS (5)

*N, numerator; D, denominator*.

Outcomes were considered both in terms of HIV infections averted and DALYs averted. They were averaged for all of the recipients receiving service from providers who were part of the intervention. The latter were calculated using the standard method for burden of disease (9, 10) (Table 4).

**Table 4:** **Sources and results for DALY calculations**.

Description	HIV with ART	HIV with no ART	AIDS with no ART	Reference
Discount rate	0.03	0.03	0.03	Assumed
Disability weight (1 for death)	0.053	0.221	0.547	Mather et al. (10), Salomon et al. (11), Comision Nicaraguense del SIDA (6), and USAID Nicaragua (4)
Age at death (YLL)	60	36	36	Comision Nicaraguense del SIDA (6) and USAID Nicaragua (4)
Life expectancy at age of death	21	42	42	Comision Nicaraguense del SIDA (6) and USAID Nicaragua (4)
Years between onset and death	30	10	2	Comision Nicaraguense del SIDA (6) and USAID Nicaragua (4)
Age at onset	26	26	26	Comision Nicaraguense del SIDA (6) and USAID Nicaragua (4)
Years with disability	30	8	2	Mather et al. (10) and Salomon et al. (11)
Years of life lost	4.58	18.88	24.00	Calculated
Years of life lost to disability	1.41	2.35	1.61	Calculated
DALYs lost	5.98	21.23	25.61	Calculated
Percent of people with HIV in group	67	33	33	Calculated
DALYs lost overall illness	4.01	7.01	8.45	Calculated

Total estimated DALY burden per case of HIV in Nicaragua			19.46	Calculated

Monte Carlo simulations were used to estimate the incremental cost-effectiveness of the PrevenSida intervention in 2014 compared to the situation for HIV prevention activities before PrevenSida began its work. The results are presented in 2014 international dollars. Given that this analysis was conducted solely from the perspective of the funder of the PrevenSida Project, we did not include the cost of treating HIV/AIDS or other medical costs associated with the changes in behavior that may be attributed to prevention messages delivered due to PrevenSida.

The expected number of HIV cases averted due to reduction in risk behavior is 100 (95% CI: 8–175), and the expected number of DALYs averted with the PrevenSida strategy was about 1,340 (95% CI: 768–1,954). The cost-effectiveness of the PrevenSida Project was estimated at $50,700 per case of HIV averted or $2,600 per DALY averted. Because of the uncertainty in the input variables, there was a 95% confidence interval between $1,000 and $99,000 per case of HIV averted and between $50 and $5,100 per DALY averted.

## Discussion

The PrevenSida Project distributed about $600,000 in grants and spent about $230,000 to provide technical and administrative assistance to 24 HIV/AIDS NGOs throughout Nicaragua in 2014. In the same year, the number of individuals considered in KPs served by NGOs involved in the project was just over 72,955, for a total cost per individual served of less than $12, which is 0.26% of the Gross Domestic Product per capita (GDPPC). In terms of efficiency, the intervention cost approximately $2,600 per DALY averted, which is a little over half the GDPPC and therefore is considered highly cost-effective according to WHO criteria for an efficient health intervention (12). The NGOs themselves organized and managed cost-sharing outside the PrevenSida mechanism, and these costs were not included in this cost-effectiveness analysis because the perspective was of the PrevenSida funder, USAID, and not the NGOs or society at large. The amount of cost-sharing was approximately $233,000 per year; more than half of this amount was the utilization of volunteer labor and the share of office expenses in situations where the NGO had negotiated shared office space in which to operate along with other organizations. This model was promoted by PrevenSida to develop a greater degree of engagement among the NGOs and to help develop a model for sustainability of the activities beyond the involvement of PrevenSida (personal communication; April 10, 2015).

Comparing the costs and efficiency in terms of spending per recipient of services, more experienced sites received a higher amount of absolute funding, but because they were providing services to substantially more individuals, they were a third to three times less costly per capita. The PrevenSida administrative costs were approximately the same per NGO receiving the technical assistance; therefore, the number of KPs the NGO provided services to was the main driver of the efficiency of their program. Given that the larger NGOs were the first to be included in the project, they look more efficient. Some of the technical assistance provided by PrevenSida was to improve management capacity in the NGO, and it was seen that fewer inputs were required over time for this type of assistance. It can be expected that if other NGOs providing services to KPs are added to the program in the future, they will appear less efficient because they will likely be serving fewer individuals in KPs and require more capacity-building inputs than NGOs already part of the project. However, both equity and efficiency issues should be addressed when implementing programs aimed at HIV services because always deferring to efficiency may lead to greater and more problematic inequities (13).

The cost and efficiency of the combination prevention model as implemented by NGOs receiving support from PrevenSida varied substantially by region, again more as a reflection of the number of people in KPs that the NGOs were providing services for. The five NGOs in the Caribbean Region served about 10,000 people in KPs using grants totaling about $134,000, while the four in the Central Region served 20% more people with 42% less in grant funding. However, the biggest difference was in the cost of providing administrative and technical support, which was three times as much in the Caribbean Region as in the Central. Again, the issue of equity versus efficiency must be considered when making decisions in light of these data.

This study had limitations, some common to economic and epidemiological modeling and some due to data deficiencies. Several assumptions were made with the cost-effectiveness model. The discount rate of 3% per year is standard in this type of analysis. It could be argued that age weighting should have been used to account for the fact that the highest incidence of HIV occurs in those who are generally the most productive and therefore have the highest DALY age weighting. Doing so would have improved the cost-effectiveness of the project; instead, we produced a more conservative estimate. We assumed that those members of KPs who received services from the NGOs cost approximately the same regardless of their age, although this may not have been the case in reality. We also assumed that the new cases averted due to the intervention would have occurred at the same average age of those who have so far contracted HIV in Nicaragua. However, it is unlikely this input would have much of a difference in the overall result. Many figures used in the cost-effective model were based on epidemiological estimates using calculations given by UNAIDS. While these are widely used in such projections, it would have been preferable to have enough follow-up time to collect actual outcome data.

## Conclusion

The technical support given by PrevenSida appears to be cost-effective by WHO standards compared to the *status quo*, and therefore we recommend that implementation of this form of capacity development be continued. While it appears to be less efficient for new NGOs that provide services to fewer people in KPs, it is still likely to be cost-effective by international standards. These findings show that such targeted capacity development interventions aimed at organizations that provide services to KPs where the HIV epidemic has the greatest effect can be acceptably efficient, at least in this setting.

## Author Contributions

EB conducted the analysis and was primarily responsible for producing the first draft. RA, AO, and ON oversaw the intervention and led all data collection, entry, and cleaning; All authors reviewed, edited and approved the final draft.

## Conflict of Interest Statement

ON, RA, and AO are employed by URC Nicaragua, which implemented the program. EB is employed by a separate project by URC Headquarters in the United States. No authors received financial benefit based on the result of the evaluation.

## References

[1] Jarquin Y Ochoa JF Larios LM Hernández C USAID Nicaragua HIV Bilateral Program Mid-Term Performance. Managua: USAID (2013).

[2] Ministerio de Salud of Nicaragua. Ministerio de Salud: Prevalencia de embarazadas sitos centinelas octubre noviembre 2011. Managua: Government of Nicaragua (2011).

[3] United States President’s Emergency Plan for AIDS Relief. PEPFAR 3.0. Controlling the Epidemic: Delivering on the Promise of an AIDS-Free Generation. Washington, DC: Department of State (2015).

[4] USAID Nicaragua. HIV Bilateral Program Mid-Term Performance: 2007–2013. Managua: U.S. Agency for International Development (2014).

[5] UNAIDS. Modelling the distribution of new HIV infections by modes of transmission. Know Your Epidemic. Geneva: UNAIDS (2014).

[6] Comision Nicaraguense del SIDA. Modelo de Modos de Transmisión del VIH: Análisis de la distribución de nuevas infecciones por el VIH y recomendaciones para prevención. Fortalecer la Respuesta Centroamericana al VIH. Managua, Nicaragua: USAID (2012).

[7] Instituto Nacional de informacion de desarrollo. Estimaciones y projecciones de poblacion nationale: departamental y municipal revicion, 2007. Managua, Nicaragua: Government of Nicaragua (2007).

[8] PrevenSIDA. In: University Research Co., editor. Sistema de registro único de poblaciones clave. Managua, Nicaragua: University Research Co. (2014).

[9] Fox-Rushby JA Hanson K. Calculating and presenting disability adjusted life years (DALYs) in cost-effectiveness analysis. Health Policy Plan (2001) 16(3):326–31. 10.1093/heapol/16.3.326 11527874

[10] Mather CD Lopez AD Murray CJ The burden of disease and mortality by condition: data, methods, and results. In: Lopez AD Mather C Ezzati DM Jamison DT Murray CJ, editors. The Global Burden of Disease and Risk Factors. Washington, DC: Oxford University Press and The World Bank (2006). p. 40–84. 21250373

[11] Salomon JA Vos T Hogan DR Gagnon M Naghavi M Mokdad A Common values in assessing health outcomes from disease and injury: disability weights measurement study for the Global Burden of Disease Study 2010. Lancet (2012) 380(9859):2129–43. 23245605 10.1016/S0140-6736(12)61680-8 PMC10782811

[12] World Health Organization. Cost-Effectiveness and Strategic Planning (WHO-CHOICE). Geneva: World Health Organization (2015).

[13] Wilson DP Kahn J Blower SM. Predicting the epidemiological impact of antiretroviral allocation strategies in KwaZulu-Natal: the effect of the urban-rural divide. Proc Natl Acad Sci U S A (2006) 103(38):14228–33. 10.1073/pnas.0509689103 16968786 PMC1599939

